# Hypertensive Heart Disease: A Narrative Review Series—Part 3: Vasculature, Biomarkers and the Matrix of Hypertensive Heart Disease

**DOI:** 10.3390/jcm13020505

**Published:** 2024-01-16

**Authors:** Valeriya Nemtsova, Annina S. Vischer, Thilo Burkard

**Affiliations:** 1 Medical Outpatient Department and Hypertension Clinic, ESH Hypertension Centre of Excellence, University Hospital Basel, 4031 Basel, Switzerland; valeriya.nemtsova@usb.ch (V.N.); annina.vischer@usb.ch (A.S.V.); 2 Internal Diseases and Family Medicine Department, Educational and Scientific Medical Institute of National Technical University «Kharkiv Polytechnic Institute», 61000 Kharkiv, Ukraine; 3 Faculty of Medicine, University of Basel, 4056 Basel, Switzerland; 4 Department of Cardiology, University Hospital Basel, 4031 Basel, Switzerland

**Keywords:** hypertensive heart disease, vasculature, circulating biomarkers, hypertension, blood pressure

## Abstract

Over the last few decades, research efforts have resulted in major advances in our understanding of the pathophysiology of hypertensive heart disease (HHD). This is the third part of a three-part review series. Here, we focus on the influence of high blood pressure on the micro- and macroalterations that occur in the vasculature in HHD. We also provide an overview of circulating cardiac biomarkers that may prove useful for a better understanding of the pathophysiology, development and progression of HHD, and may play a unique role in the diagnostic and prognostic evaluation of patients with HHD, taking into account their properties showing as abnormal long before the onset of the disease. In the conclusion, we propose an updated definition of HHD and a matrix for clinical classification, which we suspect will be useful in practice, allowing an individual approach to HHD patients.

## 1. Introduction

Over the last few decades, research efforts have resulted in major advances in our understanding of the pathophysiology of HHD. Previous parts of this review series have focused on micro- and macrostructural abnormalities of the left ventricle, left atrium and conduction system, which appear during different periods and stages of HHD development and progressing [1,2]. This third part is devoted to micro- and macrovasculopathies that may manifest alone or in combination in response to elevated BP, and the description of the role of the most studied new biomarkers in HHD. In the final conclusion of all three parts, an updated definition and a matrix of HHD for clinical classification will be proposed.

There is robust evidence for the influence of high blood pressure (BP) on micro- and macroalterations that occur in the vasculature of the heart and other target organs like eyes or kidney. Intramyocardial coronary arteries and arterioles, as well as peripheral vessels, are exposed to structural remodeling [3]. Vascular remodeling is considered a hallmark of vascular disease’s severity and progression, and is closely associated with the course and prognosis of arterial hypertension [4,5]. Like the progression of myocardial remodeling, structural alterations in the coronary microvasculature also continue to develop and are related to BP burden. Some experimental and epidemiological data support the hypothesis that the increased CV risk associated with HDD is in part due to myocardial ischemia, which can be induced by a variety of factors leading to a decreased coronary reserve [6]. The presence of LVH is one of these factors predisposing one to functional and structural changes in the myocardium, including impaired coronary hemodynamics with reduced coronary blood flow and reserve [7]. Concurrently, atherosclerosis of large arteries and increased resistance of muscular arterioles increase the afterload, leading to hypertrophy of cardiomyocytes [6].

It is well known that even patients with well-controlled hypertension may have complaints of exercise limitation or chest pain, which may not be reflected by standard resting echocardiography, stress-testing, or coronary angiography. In this regard, plasma circulating biomarkers that could identify early hypertensive patients at risk of LVH, early stages of HF, or other forms of subclinical hypertension-mediated organ damage would be useful in clinical practice [8]. There are numerous studies investigating different biomarkers in cardiovascular diseases, including hypertension, but to date, none of these biomarkers, some of which are very promising, have made it into daily clinical use in the setting of arterial hypertension [3,8]. Due to the large body of evidence related to its relevance to the context of heart failure, N-terminal-pro-B-type natriuretic peptide (NT-proBNP) is the furthest along this path, while other biomarkers are still in the earlier stages of evaluation.

Finally, the third part ends with a brief summary of all described hypertensive changes of the heart, leading to the proposal of an updated definition and a matrix of HHD combining target organ structures and clinical severity. We anticipate that this will be useful for clinical practice and avoid incomplete evaluations of changes taking place in different phenotypes of HHD.

## 2. Materials and Methods

The literature search and eligibility criteria were described in detail in part one of this review series [1]. In brief: This is a comprehensive systematic review of articles published from 1 January 2000 to 1 October 2023. MEDLINE (PubMed), EMBASE, Scopus, Web of Science, and Cochrane Central databases were searched using the following keywords and search terms: [hypertensive heart disease], [left ventricular hypertrophy], [adverse cardiac remodeling], [cardiac remodeling], [hypertension], [hypertensive heart failure], [heart failure], [myocardial fibrosis], [hypertensive cardiopathy], [cardiac biomarkers], [circulating biomarkers], [atrial fibrillation], [arrhythmia].

## 3. Review

### 3.1. General Effects of Elevated Blood Pressure on Vasculature

Vascular dysfunction and vascular remodeling are caused by chronically elevated systemic arterial blood pressure (BP), and can be associated with changes in all vessel layers, from the endothelium to the perivascular adipose tissue (PVAT) [9]. In HHD, large artery alterations are characterized by increased proximal artery enlargement, arterial wall thickness, the elongation and widening of the aortic arch, and increased arterial stiffness [5]. Small artery alterations are characterized by eutrophic remodeling (defined as increased media-to-lumen ratio with unchanged total wall tissue), increased arterial stiffness, and microvascular rarefaction [5]. It is considered that the progressive course of HHD leads to a reduction in the microcirculatory network, and many bioactive molecules, such as angiotensin-II (Ang-II), endothelin-1 (ET-1), aldosterone, catecholamines, and metalloproteinases (MMP) have an impact on vascular remodeling, particularly because of their capacity to increase oxidative stress and to impair nitric oxide (NO) activity in the vascular wall [4,5].

#### 3.1.1. Mechanisms and Clinical Role of Arterial Remodeling in Hypertensive Heart Disease

Vascular remodeling is classified as hypertrophic, eutrophic, or hypotrophic [9,10]. Remodeling can also be inward (reduced luminal diameter) or outward (increased luminal diameter) [9]. Inward remodeling is the most common type of vascular remodeling in hypertension (HTN), causing a reduction in the luminal diameter under passive conditions, and outward remodeling is generally seen during antihypertensive treatment [9].

#### 3.1.2. Peripheral Vascular Resistance

Increased peripheral resistance, predominantly observed in small blood vessels, is one of the most distinctive features of HTN. The functional and structural alterations of small resistance vessels that occur either before or as a result of chronically elevated BP in their turn intensify the effects of vasoconstrictors (ex. of the renin–angiotensin–aldosterone system, RAAS) [11]. In HTN, increased peripheral resistance is because of structural rather than functional changes in the resistance vasculature. This was first demonstrated in the 1950s by the work of Folkow, and was later supported by animal studies, which suggested that the morphological changes seen in HTN are similar in different vascular beds [10,12,13].

#### 3.1.3. Arterial Stiffness

Classically, there are two distinct components of BP: mean arterial pressure (MAP) and pulse pressure (PP) [14]. MAP reflects a steady pressure, related to vascular resistance and hence small arteries, whereas PP reflects a pulsatile pressure, which has three determinants, stroke volume, arterial stiffness and wave reflections [14]. Arterial stiffening is defined as resistance to deformation or a loss of elastic compliance due to changes in the geometry and microstructure of the vascular wall [15].

Arterial stiffness, as well as wave reflections, have continued to be a focus of investigations in HTN since the 1970s [11]. In clinical studies and animal models, it was clearly demonstrated that in addition to increased vascular resistance, vascular elasticity was also consistently impaired in HTN, thus indicating the presence of increased stiffness in large artery walls [11,16]. It is well known that arterial stiffness is one of the earliest features of adverse structural and functional changes within the arterial wall, and is recognized as an independent predictor of cardiovascular (CV) mortality in patients with essential HTN [17,18]. Notably, in recent years, findings from animal- and population-based studies support the notion that arterial stiffness may be not only a complication of HTN, but also a risk factor for the development of high blood pressure [5].

The mechanical properties of large elastic blood vessels, including elasticity and the ability to store energy during deformation, which is essential for peripheral perfusion during diastole, are altered and reduced by vascular wall stiffening, leading to an increase in cardiac afterload [15,19]. Additionally, the increased forward propagation of pulsatile waves transmits pressure oscillations to end-organs, which fosters damage to the microcirculation and promote CV morbidity and mortality [15].

#### 3.1.4. Cardiac Microvasculature and Hypertensive Heart Disease

Myocardial ischemia in HHD can be induced by a variety of factors, relates to the degree of increase in left ventricular (LV) mass and has important clinical implications [6]. At the beginning of the development of HHD, coronary flow may be increased because of higher BP, greater LV end-systolic stress, and LV hypertrophy (LVH). On the one hand, the presence of LVH could be associated with decreased vascular density, which seems to result from inadequate angiogenesis. The direct compression of the endocardial capillaries can result as a response to the increasing muscle mass [6,20]. Breisch and colleagues studied the effects of pressure overload hypertrophy in the LV myocardium at different times after constriction of the aorta in adult cats, and found that the capillary density and coronary reserve decreased with the increasing extent of hypertrophy. The authors assumed that such alterations in flow reserve and capillary density might play an important role in the transition from a compensated to a failing heart [6,21]. Tomanek et al. also showed that late-onset HTN in middle-aged and senescent rats is characterized by microvascular alterations, including decrements in numerical density and the inadequate growth of capillaries that reflect an absolute reduction in the number of these vessels in the context of the presence of cardiocyte hypertrophy [22]. There is an assumption that alterations in pericytes, which surround capillary endothelial cells, may also contribute to the paucity of microvessels in HHD [20]. On the other hand, increased perivascular fibrosis is accompanied by an increase in oxygen diffusion distance, leading to the impairment of oxygen supply to cardiomyocytes [7,20,23]. Of note, impaired endothelium-mediated dilation, first of all of the resistance coronary arteries, may play an important role in abnormal coronary blood flow regulation, even in early stages of coronary atherosclerosis, which is commonly encountered in HTN [18], and could lead to chronic subendocardial ischemia and impaired myocardial mechanical function [19,24]. The presence of ongoing cardiac ischemia may also explain the increased CV risk, including the increased incidence of atrial fibrillation, ventricular arrhythmias, myocardial infarction and sudden cardiac death, attributed to HHD [2,25].

#### 3.1.5. Determinants and Evaluation of Coronary Microcirculation

Currently, no widely available technique allows for the direct visualization of the coronary microvasculature in vivo in humans [20]. The function of coronary microcirculation can be indirectly assessed using coronary flow reserve (CFR) in response to various vasoactive stimuli. CFR is an integrated measure of flow through both the large epicardial arteries and the coronary microcirculation. In the absence of an obstructive stenosis of the epicardial arteries, reduced CFR is considered to be a biomarker for coronary microvascular dysfunction [20,23], and could possibly help to explain the phenomena of silent ischemia, chest pain, and coronary insufficiency in HHD, especially without angiographic signs of coronary stenosis [18,26]. 

It should be noted that in hypertensive animal models and patients with HHD and LVH, perivascular fibrosis together with endothelial dysfunction may contribute to impaired CFR by the external compression of intramural coronary arteries [20]. However, interestingly, Vancheri FT et al. indicated that the impairment of CFR in hypertensive patients is independent of the presence and degree of LVH [24]. Reduced diastolic myocardial perfusion pressure because of increased arterial stiffness also contributes to CFR impairment in hypertensives. Additionally, perivascular fibrosis has been shown to be inversely correlated with CFR in heart failure (HF) [23,27]. Several observations have found early alterations in CFR and LV diastolic function in arterial HTN, including borderline HTN, even before the development of LVH [26]. On the other hand some studies in experimental models of HTN as well as in hypertensive patients came to the conclusion that resting coronary blood flow may—even in the presence of LVH—be normal [26].

A new index of the coronary vasodilatory capacity—the microvascular resistance reserve (MRR)—is now recommended [28]. It was introduced to characterize the vasodilator reserve capacity of the coronary microcirculation while accounting for the influence of concomitant epicardial disease and the impact of vasodilators administration on aortic pressure [28]. Boerhout and colleagues concluded, with reference to the global ILIAS (Inclusive Invasive Physiological Assessment in Angina Syndromes) Registry data, that MRR is a robust indicator of the microvascular vasodilator reserve capacity, and is significantly associated with major adverse cardiac events (MACE) at 5-year follow-up in vessels with functionally significant epicardial disease [29].

Recent studies using the positron emission tomography (PET)-derived flow/mass ratio showed the prevalence of coronary microvascular dysfunction in the absence of obstructive epicardial coronary artery disease in HTN and HF with preserved ejection fraction (HFpEF) [30,31]. The authors of this study concluded that subendocardial ischemia is one of the central pathways in HFpEF pathogenesis, and that higher troponin levels at rest and during exercise, in combination with coronary microvascular dysfunction, serve as identification criteria for patients with HFpEF at especially high risk for adverse CV outcomes [30]. Thus, the inability of myocardial perfusion to meet the myocardial oxygen demand may be a potential explanation of adverse CV outcomes in patients lacking angiographically confirmed obstructive coronary artery disease, in particular in pathological patterns of LV remodeling [30]. 

It is considered that the progression from initial changes in HHD to hypertensive HF cannot be explained only by the myocardial response to elevated BP; both coronary and peripheral vasculopathies are assumed to play an essential role [24]. However, the full extent of their contribution to this process is still uncertain. In addition to the hemodynamic effects of chronically elevated BP on LV wall stress, cardiomyocyte hypertrophy and coronary artery stiffening, as well as non-hemodynamic and cardiometabolic factors, may contribute significantly to the impaired myocardial perfusion and resultant HHD development [30]. Future investigations of the coronary microvascularisation should include the mechanisms by which ventricular perfusion adapts to meet the functional demands imposed by the hypertensive ventricle, and can facilitate risk assessment in HHD [30].

#### 3.1.6. Effect of Hypertensive Heart Disease on the Pulmonary Vasculature

The evaluation of pulmonary vascular remodeling, including increased arterial wall thickness and alveolar wall remodeling, in chronic HTN and HF has received considerable attention in the last decade [32]. The sustained elevation of pulmonary capillary pressure, resulting from recurrent retrograde increases in LV enddiastolic filling pressure in patients with chronic HF, affects the capillary diffusion efficiency and associated gas exchange [33]. Additionally, as a result of the increased activation of neurohumoral mediators leading to myofibroblast proliferation with collagen and interstitial matrix deposition, excessive alveolar wall thickening is responsible for the development of a restrictive lung syndrome. As result, compliance is reduced and gas exchange impaired, contributing to shortness of breath and pulmonary hypertension (PH) in HF patients [33,34,35]. Furthermore, hypoxia promotes vasoconstriction in the pulmonary circuit and tachyarrhythmias, particularly atrial fibrillation, which may precipitate PH in patients with HF [36]. Over time, pulmonary arteriolar remodeling mainly contributes to the increase in pulmonary vascular resistance and reduced pulmonary artery compliance [36].

Farrero et al. showed that, in adult patients with HFpEF and hypertensive controls, endothelial dysfunction and abnormal collagen metabolism can be characterized as PH risk markers [37].

Despite significant recent developments in our understanding of the pathophysiology of PH associated with hypertensive HF, important evidence gaps remain as regards the targeting of an ideal approach to the management of hypertensive and HF patients developing PH.

#### 3.1.7. Clinical Significance of Peripheral Arterial Remodeling and Cardiovascular Risk in Hypertensive Heart Disease

Over the past few years, arterial stiffness and wave reflections have been widely investigated in hypertensive subjects. It was shown that not only systolic BP and diastolic BP, but also PP is an independent marker of CV risk in hypertensive subjects, especially those with recurrent myocardial infarction and congestive HF [38].

Observational studies have shown that the extent of arterial remodeling is linked to clinical outcomes [5]. As a prominent example, in the Framingham Heart Study (FHS), greater arterial stiffness (assessed by carotid-femoral pulse wave velocity, PWV) was associated with increased risk of a first CV event [39]. Similarly, a recent meta-analysis of 17,635 participants showed that aortic stiffness, assessed by measurement of aortic PWV, was associated with a 30% higher risk of cardiovascular diseases (CVD), and predicts future CV events and mortality, even after accounting for other established CV risk factors [5,40]. In the population-based Rotterdam Study, it was found that among apparently healthy normotensive subjects, arterial stiffness independently predicts stroke and coronary heart disease [41]. Long-term longitudinal studies have shown that elevated PP leads to an approximately two-fold increase in CV risk, whereas MAP represents a significantly lower component [42,43]. Aortic PP is considered as a more reliable parameter for the evaluation of CV risk than brachial PP [11].

Blacher J et al. showed, based on the pooled results of three placebo-controlled trials in elderly patients with HNT (the European Working Party on High Blood Pressure in the Elderly trial (EWPHE), the Systolic Hypertension in Europe Trial (Syst-Eur), and the Systolic Hypertension in China Trial (Syst-China)), that increased PP, but not MAP, is a powerful predictor of CV risk in older hypertensive patients [43].

#### 3.1.8. Vasculopathy in Hypertensive Heart Disease as Therapeutic Target

Humans and hypertensive animal models studies, as well as epidemiological studies, have shown that both small and large arteries are primary targets for antihypertensive therapy [11,44,45]. Studies investigating the effects of angiotensin-converting enzyme (ACE) inhibitors, angiotensin II receptor blockers, and selective aldosterone antagonists have concluded that BP reduction alone causes the regression of aortic wall hypertrophy, whereas the aortic collagen content is affected by factors that are independent of BP lowering [11,44]. In addition, AT1 or mineralocorticoid receptor blockade against the background of a low-sodium diet also reduced wall stiffness, independently of changes in BP [11,44,45].

The REASON project (preterax in regression of arterial stiffness in a controlled double-blind study), a multicenter, randomized, double-blind, two-parallel-group study, which included 471 adult hypertensive patients, showed that a combination therapy with perindopril plus indapamide improved arterial stiffness and reduced PP amplification, whereas atenolol reduced heart rate, but did not affect PWV [46]. Additionally, the CAFE (Conduit Artery Function Evaluation) study confirmed the superiority of a stable amlodipine ± perindopril regimen over atenolol ± thiazide-based treatment in lowering central aortic pressure in hypertensives, and underlined the importance of central aortic PP in terms of clinical outcomes [47]. However, a meta-analysis of individual data from seven short-term (less than 4 weeks) and nine long-term (4 weeks and more), double-blind, randomized therapeutic trials, conducted by Ong KT et al., showed that in long-term trials, ACE inhibitors, calcium antagonists, β-blockers, and diuretics all significantly reduced arterial stiffness beyond BP reduction in essential hypertensive patients, whereas in short-term studies, the decrease in arterial stiffness was less under calcium channel blockers (CCB) treatment than under ACE inhibitors [48]. 

The interesting effects of sacubitril/valsartan compared with olmesartan were noted by Schmieder et al. in a double-blind, randomized study of hypertensive patients [49]. Against the background of reductions in LV mass, measured by MRI, and central pulse pressure, there was no significant difference in local distensibility between the two groups for the short-term period of follow-up (12 weeks) versus the long-term period (52 weeks) of follow-up [49].

The reversibility of arterial stiffness and wave reflections in response to drug treatment raises many questions related to the CV risk reduction and the treatment of HTN. Improvements in both, beyond lowering blood pressure, would be a promising aspect of further risk reduction in the treatment of hypertensive patients, and should be a focus of further research.

### 3.2. Neurohumoral Changes and Biomarkers

The role of circulating biomarkers in the context of myocardial remodeling, myocardial fibrosis and the extracellular matrix has been reviewed and discussed in detail in part one of this review series [1]. Additionally, the role of circulating N-terminal pro-brain natriuretic peptide (NT-proBNP) was in part discussed in the context of HHD and HF in part two [2]. Therefore, we will focus in this part on the biomarkers in the context of HHD that are already in clinical use or close to implementation.

According to the European Society of Cardiology Working Group on Peripheral Circulation, at present, a biomarker should satisfy such criteria as proof of concept, prospective validation, incremental value, clinical utility, clinical outcomes, cost-effectiveness, ease of use, methodological consensus, and reference values [50]. There are well-known biomarkers used for the diagnosis and management of several causes of secondary HTN (plasma aldosterone to renin ratio (ARR), metanephrines, cortisol, etc.). The selected cardiac biomarkers are recommended as factors identifying individuals that are at higher CV risk, or are useful in the diagnosis and management of several cardiac conditions, and include lipoprotein (a), high-sensitivity cardiac troponin T (hs-cTnT) and high-sensitivity cardiac troponin I (hs-cTnI), brain natriuretic peptide (BNP) and NT-proBNP [51]. In HTN, all biomarkers can be divided into groups reflecting CV dysfunction, inflammation and oxidative stress. They can also be evaluated as predictors of HTN development and prognosis [52]. However, at present, no single biomarker can give a full picture of the pathophysiological process. It seems to be impossible to clearly identify a specific pathway that leads to changes in a particular marker’s concentration solely related to HHD. However, the correlations and causal interactions identified in different studies allow us, to some extent, to assess the specific impact of a particular marker on different HHD patterns. The 2023 European Society of Hypertension guidelines emphasize the potential role of circulating biomarkers in the detection of early functional and structural cardiac changes associated with HTN, including primary care patients [51]. The roles of endothelin, NO and RAAS molecules, and the balance between NO and Ang-II levels as one of the central aspects in the pathogenesis of HTN, have previously been intensively investigated and described [6,53,54,55]. However, despite intensive research, the role of circulating cardiac biomarkers in the diagnosis, progression and prognosis of HHD remains controversial and unspecified.

**Figure 1:**
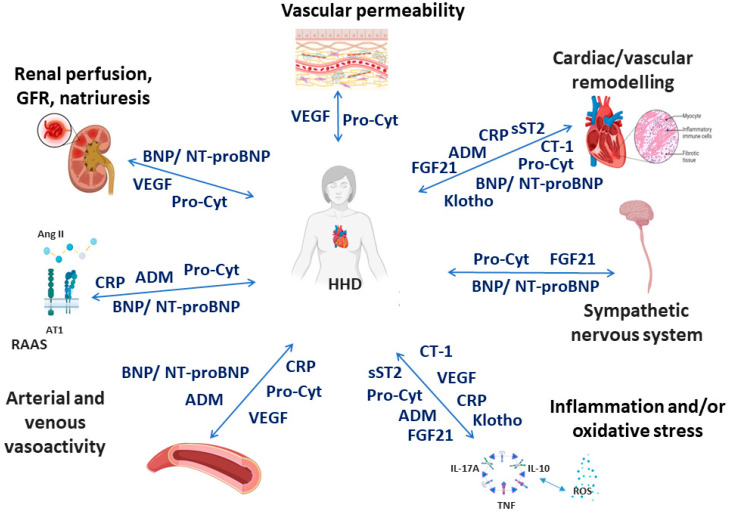
Simplistic and schematic representation of the association between selected biomarkers and pathophysiological cardiovascular effects in hypertensive heart disease. AII—angiotensin II, ADM—adrenomedullin, AT1—angiotensin 1 receptor, BNP—brain natriuretic peptide, CRP—C-reactive protein, CT-1—cardiotrophin-1, FGF21—fibroblast growth factor-21, GFR—glomerular filtration rate, HHD—hypertensive heart disease, NT-proBNP—N-terminal pro-brain natriuretic peptide, Pro-Cyt—proinflammatory cytokines, RAAS—renin-angiotensin-aldosterone system, sST2—soluble suppression of tumourigenicity 2, VEGF—vascular endothelial growth factor. Created with Biorender.com. accessed on 8 December 2023.

The following biomarkers were selected based on the most recent data, suggesting their potential utility for clinical use in the context of HHD (Figure 1). Most of these biomarkers play a role in the development and progression of adaptive and maladaptive processes in the heart and blood vessels in HHD [1,2]. However, some of them are considered as markers of a variety of physiological and pathophysiological processes.

#### 3.2.1. Brain Natriuretic Peptide and Its Derivatives

At the end of twentieth century, the natriuretic peptides (NPs) family was described as consisting of atrial natriuretic peptide (ANP), brain natriuretic peptide (B-type NP, BNP,) and C-type natriuretic peptide (C-type NP, CNP) [56,57]. The cardiac NPs system (ANP and BNP), due to its diuretic, natriuretic, vasorelaxant, cardiac and vascular antifibrotic and antihypertrophic effects, RAAS and sympathetic nervous system inhibition, as well as anti-atherosclerotic properties, appears to be a key candidate among potential biomarkers involved in the pathogenesis of HTN and HHD (Table 1) [56,57,58]. NPs have been shown to be a strong independent marker of new-onset HF and other CVD [57]. Changes in NP levels over time in patients with chronic HF predict not only the risk of adverse outcomes, but also predict changes in LV size and function [57]. There data suggest that the cardiac NPs system may also play a role in protecting the heart against hypertrophy [56]. The International Consortium for Blood Pressure Genome-Wide Association Studies performed a study in both Caucasian and Asian populations, and found that the genes for BNP and ANP are among the most relevant ones in determining risk of HTN [59,60]. The gene for BNP was identified as one of the most relevant in HTN, while none of the RAAS genes have been identified as contributing to the polygenicity of HTN [56]. Studies on genetic variants of NP genes have supported the existence of a genetic predisposition to HTN that is characterized by lower circulating levels and/or activities of cardiac NPs [56]. This is consistent with the findings of Macheret et al., who found reduced levels of BNP1–32 and decreased activation of its precursor NT-proBNP1–76 in prehypertension and early stages of HNT in a general population of 2082 adults [58]. They also found a tendency towards elevation in these peptides only in stage 2 HTN, with no compensatory increase in ANP in advanced HTN [58]. This study partly confirms the results of an earlier study by Belluardo P et al. who also found a lack of activation of BNP32 together with a reduction in NT-proBNP in early stages of HTN, and an increase in more advanced stages [61]. The finding of such a deficiency of biologically active NPs, despite the increase in BNP levels in later stages of HTN, suggests that this is probably a late compensatory effect, as it correlates with the severity of HTN [56,61]. 

This somewhat contradicts the evidence for increased BNP production as a marker of LV dysfunction [62]. Experimental studies have shown that even in the initial stages of ventricular hypertrophy, and especially in those with LVH, plasma BNP levels were higher than in normotensive subjects [62]. There is experimental evidence that even small elevations in NT-proBNP may be indicators of adverse remodeling on the pathway from LVH to clinical HF [63]. Moreover, several studies have shown that elevated NT-proBNP, together with troponin T or hs-cTnT, may identify patients with LVH at higher risk of HF and death [63,64,65]. Neeland et al. found a two-fold higher NT-proBNP level in those with LVH compared to those with a normal LV geometry in the general population, and higher levels were associated with more severe LVH [63]. Seliger and colleagues demonstrated analogous results in the prospective observational study of CV risk factors in older adults, the Cardiovascular Health Study (CHS), by analyzing NT-proBNP levels at baseline and after 2 to 3 years in older adults without prior HF or myocardial infarction [64]. It was found that baseline biomarker elevation appeared to be more associated with an increased risk of progression to HF with reduced ejection fraction (HFrEF) than HFpEF in those with LVH [64]. In addition, the authors demonstrated an approximately three-fold higher risk of developing HF, primarily HFrEF, in those with LVH and longitudinal increases in NT-proBNP, compared with those without LVH and with stable biomarkers [64].

On the other hand, Ojji and colleagues found no difference in NT-proBNP levels between hypertensive subjects with LVH and those without LVH [8]. Furthermore, the authors of this study showed no correlation of NT-proBNP with LV mass index, interventricular septal wall thickness and LV posterior diastolic wall thickness, but found a significant correlation with MAP, PP and age [8].

Of note, in a cohort study that included 684 patients, it was shown that plasma NT-proBNP is a powerful prognostic indicator of mortality in HTN, superior to and independent of ECG markers, namely, the Sokolov index and the RaVL amplitude [66]. 

**Table 1:** Potential pathogenetic, clinical and prognostic role of selected biomarkers in the setting of hypertensive heart disease.

Biomarker	Role in the Pathogenesis of HHD/HTN	Prognostic Role	Clinical Potential	Possible Limitations
B-type NP/NT-proBNP	Diuretic, natriuretic, vasorelaxant, cardiac and vascular antifibrotic and antihypertrophic effects, RAAS and SNS inhibition, antiarrhythmic [56,67]	Indicator of adverse LV remodeling; NT-proBNP with troponin T or hs-cTnT may identify patients with LVH at higher risk of HF and death; associated with severity of LVH	Associated with the development of HTN [8];c orrelates with the severity of HTN; improves risk stratification [8], predictors of AF onset and progression	The additive effect of age and obesity on B-type NP levels
ADM/MR-proADM	Anti-inflammatory, vasodilatory, antiproliferative and antioxidant effect, RAAS inhibition [68,69]	Higher levels are related to worse prognosis in HF [68,69]	Correlates with BP, the severity of HMOD, the level of BNP	There are limited data on the prognostic role of ADM in HTN/HHD
CRP/hsCRP	Inhibits vasodilatation, increases oxidation, and upregulates RAAS and pro-inflammatory effect [70,71]	High levels of CRP in normotensive patients have predicted the development of HTN on follow-up [70]	A marker of systemic inflammation; high levels are significantly associated with an increased risk of developing HTN and its progression	Lack of specificity for the development of HTN/HHD
Pro-inflammatory cytokines	Increase vascular permeability, thrombogenesis, myocardial fibrosis, systemic inflammation and SNS activity [72,73]	IL-6 is considered an important cardiovascular risk biomarker; increased IL-6 and TNF-α serum levels proposed as independent risk factors for the development of high BP in apparently healthy patients [73]; promote endothelial dysfunction, an early predictor of atherosclerosis and cardiovascular events and mortality [73]	Associated with reduced eGFR [73]; may play a role in HMOD (LV remodeling—fibrosis, hypertrophy and dilatation) [73,74]	Lack of specificity for the development of HTN/HHD
Vascular endothelial growth factor (VEGF)	Promote vascular remodeling and abnormal angiogenesis, correlated with pro-inflammatory cytokines [75,76]	High concentrations associated with poor prognosis and CVD disease severity; may be a useful biomarker for the early detection of microvascular damage, endothelial dysfunction [75]	Elevated serum levels positively correlated with pro-inflammatory cytokines, increased BP, uncontrolled HTN and CRP, and negatively with GFR [77]	Lack of the significant data for the evaluation of risk and prognosis for essential HTN or HHD
Soluble suppression of tumourigenicity 2 receptor (sST2)	Pro-fibrotic, inflammatory effect [78]	A predictor of LV systolic dysfunction, mortality and progression in HF [78,79]; predicts future risk of death, HF, and overall cardiovascular events in apparently healthy individuals [80]	Correlated with LVH, HF severity, LVEF, creatinine clearance, BNP and CRP, associated with BP [78]; a marker of myocardial fibrosis and remodeling	Values increased with age, male sex [80]; knowledge of mechanisms regulating sST2 production in healthy subjects and in HTN/HHD patients is still scarce
Cardiotrophin-1 (CT-1)	Pro-fibrotic, pro-oxidative, pro-inflammatory effects [8,81]	Has predictive power in LVH regression in patients with mild to moderate HTN [51]	Associated with the progression of HF in patients with HTN [81]; sensitive to LVH detection [8]	Role in the development and maintenance of LVH is controversial
Fibroblast growth factor-21 (FGF21)	Alleviates oxidative stress, inflammation; improves resistance to oxidative stress; anti-fibrotic effect [82], activates SNS [83]	An independent risk factor for AF [83]; could be used as a biomarker of metabolic dysregulation; may be indicative of a higher risk of cardiovascular events [83]	Significantly associated with elevated BP in HTN [84]; showed cardioprotection in cardiac tissue [82]	Lack of specificity for the development of HTN/HHD or prognosis
Klotho	Antioxidative effect, endothelium protection; anti-proliferative and anti-inflammatory effects [85]	Independently associated with CVD [86]	Indeficiency prone to LVH, higher plasma levels associated with a lower risk of CVD and protective effect on the cardiovascular system [86]	Lack of data on specificity and the role in the pathogenesis of HTN

Note: ADM—adrenomedullin, AF—atrial fibrillation, BP—blood pressure, B-type NP—brain natriuretic peptide, CRP—C-reactive protein, CVD—cardiovascular disease, eGFR—estimated glomerular filtration rate, HF—heart failure, HHD—hypertensive heart disease, HMOD—hypertension-mediated organ damage, hsCRP—high-sensitivity C-reactive protein, hs-cTnT—high-sensitive cardiac troponin T, HTN—hypertension, IL-6—interleukin-6, LVEF—left ventricular ejection fraction, LVH—left ventricular hypertrophy, MR-proADM—mid-regional pro-adrenomedullin, NT-proBNP—precursor N-terminal-prohormone brain natriuretic peptide, RAAS- renin-angiotensin-aldosterone system, SNS—sympathetic nervous system TNF-α—tumour necrosis factor-α.

Based on these data, plasma NT-proBNP has been recommended for use in primary care for hypertensive patients, especially those without ECG evidence of LVH, to improve risk stratification [8,66]. However, data on the prognostic value of NPs in HTN are limited, and it is necessary to consider the additive effect of age on BNP levels and the influence of obesity on NT-proBNP levels that could decrease the value of this peptide as a risk marker for hypertensive CV events [66,81]. At present, NT-pro BNP is one of the most widely studied circulating biomarkers in HHD [8].

#### 3.2.2. Adrenomedullin

Adrenomedullin (ADM) is a novel cardiovascular peptide, first discovered in 1993 in the tissue of a human pheochromocytoma. It has many functions including tissue repair, anti-inflammatory and antioxidant properties.

The effects of ADM on the CV system have been extensively studied. ADM is released from the vascular wall and acts as an autocrine or a paracrine hormone to regulate vascular tone and blood pressure. It has a potent vasodilatory effect on the systemic circulation reducing BP, and also increasing blood flow in the cerebral, pulmonary and renal vasculatures [87]. Apart from the vasodilatory effect, ADM protects CV tissues from injury by inhibiting apoptosis and regulating proliferation. In addition, it has antiproliferative effects in vascular smooth muscle cells and inhibits the RAAS [87,88]. 

ADM is elevated in patients with HF, and higher levels correspond to more advanced HF and worse prognosis [88,89]. Several pre-clinical and human studies have established the possible effects of the exogenous administration of ADM, with the result of attenuating the progression of LV dysfunction and improving survival in HF [88,90].

Previous studies have shown that plasma ADM levels are higher in patients with essential HTN compared to normotensive subjects, and increase with the stages of HTN. Levels correlate not only with BP and the severity of hypertensive-mediated organ damage, but also with the levels of ANP and BNP [87,91,92]. 

Despite the fact that the exact relationship between plasma ADM level and blood pressure has not been completely confirmed, there is accumulating evidence that ADM can be suggested for clinical use as a prognistic biomarker for CVD. Nishida and colleagues compared the predictive power of plasma ADM with adiponectin and high-sensitivity C-reactive protein (hsCRP) for future CV events in high-risk patients, and indicated that ADM is superior to both [68].

In another study, a part of the ADM precursor peptide, mid-regional pro-adrenomedullin (MR-proADM), demonstrated a strong prognostic value for mortality and morbidity in patients with HF or LV dysfunction after an acute myocardial infarction [69]. Moreover, MR-proADM showed a better predictive value than BNP and NT-proBNP in this study [69]. It was shown that MR-proADM is more stable than ADM, and therefore, some authors recommend it as a more suitable biomarker for clinical use [69]. To date, the true value of ADM and MR-proADM in the special context of HHD is open, and remains to be determined.

#### 3.2.3. C-Reactive Protein and High-Sensitivity C-Reactive Protein

There is notable evidence that inflammation is involved in vascular remodeling in HTN, and is often present in the early stages of HTN [93]. C-reactive protein (CRP) is the circulating biomarker related to vascular wall biology, with many studies supporting its clinical use for further risk stratification in the setting of primary and secondary CVD prevention [50]. Present data suggest that potential mechanisms for the relationship between BP and CRP may include a quenching of nitric oxide production by endothelial cells resulting in an indirect inhibition of vasodilatation, an increased leukocyte adhesion, platelet activation, oxidation, and thrombosis, as well as an upregulation of receptor-mediated angiotensin 1 events in vascular smooth muscle cells [93]. Moreover, high CRP levels in normotensive subjects at baseline predicted the development of HTN during follow-up [52]. Blake and colleagues concluded, after examining the relationship between blood pressure, CRP and the occurrence of first CV events among 15,215 women followed prospectively over a median of 8.1 years, that CRP and blood pressure are independent determinants of CV risk, and that their predictive value is additive [93]. Despite some limitations, an interesting finding shown by Wang et al. in a prospective, large and well-defined cohort of normotensive individuals investigating the association of nine biomarkers (CRP, fibrinogen, plasminogen activator inhibitor-1 (PAI-1), aldosterone, renin, BNP, NT-proBNP, homocysteine and urinary albumin/creatinine ratio (UACR)) with the development of HTN was that the three best-performing biomarkers were CRP, PAI-1 and UACR [94]. Sesso et al. also found in a prospective study consisting of 525 females with a median follow-up period of 7.8 years that higher CRP levels were significantly associated with an increased risk of developing HTN, including those with low baseline BP levels and with no CV risk factors [70]. However, a prospective case–control study in a male population, conducted by the same authors, found no such associations [95]. Conversely to the findings of the Multi-Ethnic Study of Atherosclerosis (MESA) an association of HTN with higher CRP levels was detected in both men and women [96].

Because CRP is not detectable at very low levels, hsCRP is recommended for measurement to enable the earliest detection of a pro-inflammatory state. HsCRP is a marker of systemic inflammation that is upregulated in this context as a consequence of vascular disease, and its levels are correlated with traditional risk factors (systolic BP, lipids and body mass index), as well as other markers of inflammation, such as white blood cell count, interleukin (IL)-6 and fibrinogen levels [50]. However, as it is an acute phase reactant, elevated hsCRP levels lack specificity for CV disease [50]. 

According to the ESC guidelines for CV disease prevention in clinical practice 2015, the role of hsCRP in predicting future CV events has been established in large trials and meta-analyses, but hsCRP levels should only be measured as part of refined risk assessment in patients with moderate risk profiles, not in asymptomatic low-risk or high-risk individuals [50].

Furthermore, there are studies that have failed to demonstrate a predictive role of CRP in the development of HTN, and elevated hsCRP levels lack a specific association with CV disease [50,97,98].

#### 3.2.4. Pro-Inflammatory Cytokines

There is substantial experimental evidence that the increased vascular permeability, thrombogenesis, myocardial fibrosis, and diastolic dysfunction associated with HTN may be due to chronic inflammation [99]. Recent evidence clearly shows that inflammation not only determines the development and/or progression of HTN, but also plays an important role in the pathogenesis of hypertension-mediated target organ damage, with evidence pointing to increased inflammatory mediators even in prehypertensive patients [20,73,74,99]. Kockskamper et al. emphasize that the mediators of inflammation are of cellular and humoral nature, and both types may be circulating or locally active [100]. Findings from experimental models of HTN and hypertensive patients have shown increased plasma levels of proinflammatory cytokines, such as IL-6, IL-8, IL-23, IL-1β, IL-17A and tumour necrosis factor-α (TNF-α), or an increased presence of activated monocytes in the circulation, and are consistent with the potential existence of low-grade inflammation [20,73,99]. Serum uric acid levels have also been found to be a useful marker of inflammation and oxidative stress in HTN, and have been shown to be significantly associated with HTN and its CV complications [101].

It is important to note that elevations in inflammatory markers have not been observed by all the authors in isolated HTN; some of them have indicated that chronic inflammation becomes more evident in the presence of hypertension-mediated target organ damage [74,81]. 

The close relationship between inflammation and HTN has also been emphasized in many studies with conventional CV drugs (CCB, RAAS inhibitors, statins), which have shown additional anti-inflammatory effects that could also be related to their antihypertensive properties [73,102,103,104]. In addition, some anti-inflammatory drugs, such as mycophenolate mofetil, have been shown to reduce blood pressure or attenuate the HTN development in animal models and hypertensive patients [73,105]. 

Further studies are needed to precisely elucidate the role of proinflammatory cytokines in arterial hypertension and to evaluate the predictive power of these markers for HHD outcomes. Moreover, whether this inflammation is a consequence or a cause of HTN remains unclear.

#### 3.2.5. Vascular Endothelial Growth Factor

Vascular endothelial growth factor (VEGF) is known to be a multifunctional peptide capable of inducing receptor-mediated endothelial cell proliferation and angiogenesis [75]. There is evidence this peptide also associates with coronary artery disease, peripheral vascular disease, and acute HF, and it could be a predictor for poor prognosis in acute coronary syndrome [75]. 

In HTN, it has been shown that VEGF levels are often increased, promoting vascular wall remodeling and suggesting a contribution of abnormal angiogenesis to the pathogenesis of HTN and hypertension-related complications [106,107,108]. Tsai et al. demonstrated significantly higher VEGF levels in patients with HTN and retinopathy compared to normotensive subjects, and suggested that plasma VEGF levels may be a useful biomarker for the early detection of microvascular damage [75]. Plasma VEGF has also been shown to be a useful early marker of endothelial dysfunction, even before active intravascular inflammation had developed [75,106]. A clinical pilot study by Belgore et al. showed a significant reduction in plasma VEGF under the influence of 2 months of antihypertensive therapy [108].

In cancer patients, the use of VEGF inhibitors (VEGFi) leads to a treatment-associated increase in blood pressure and VEGFi-associated hypertension that may limit anti-cancer treatment [109,110]. 

To date, there are no recommendations to measure VEGF routinely in the evaluation of essential HTN or HHD.

#### 3.2.6. Soluble Suppression of Tumourigenicity 2 Receptor

The soluble suppression of tumourigenicity 2 (ST2) receptor is a member of the Toll-like/interleukin-1 receptor family, and is a novel circulating biomarker that has been studied and tested in differentiating the various spectra of HHD [8]. ST2 exists in two forms—transmembrane and soluble (sST2) forms. Soluble ST2 is being investigated as a candidate biomarker in different CVD entities like myocardial infarction or HF. Soluble ST2 levels have been shown to predict outcomes in patients with HF, and a change in sST2 over time is associated with a worse prognosis [8]. In a study of 210 black hypertensive patients, higher sST2 levels were observed in patients with HF compared to those without HF, regardless of the LVH presence [8,111]. Additionally, hypertensive patients with LVH had higher levels of sST2 than those without LVH. Same authors also found in a cohort of hypertensive patients without HF (n = 133) that patients with concentric LVH had the highest concentration of sST2 compared to normal LV geometry, concentric remodeling, and eccentric LVH [8,112]. In the Framingham Heart Study, sST2 was also found to be associated with BP [78].

In addition, hypertensive adult patients with an LV ejection fraction (LVEF) >50% showed significantly elevated sST2 concentrations when stable HF was present compared to hypertensive controls without signs or symptoms of HF, but without a clear association to New York Heart Association (NYHA) classification [113].

Several other studies have reported a correlation of sST2 levels with HF severity, LVEF, creatinine clearance, BNP, and CRP, or noted sST2 levels as a predictor of mortality [8,114].

Thus, sST2 may be a marker of the vascular effects associated with increased afterload and myocardial stress; however, further studies investigating an incremental value, e.g., over BNP or echocardiography in the context of LVH evolution and HHD, remain to be undertaken. To date, there are no recommendations to measure sST2 routinely in the evaluation of essential HTN or HHD.

#### 3.2.7. Cardiotrophin-1

Cardiotrophin-1 (CT-1) is a member of the IL-6 cytokine superfamily. In HTN, LVH and HF CT-1 levels are higher compared with controls [115]. The highest plasma CT-1 concentrations have been reported in the subgroup of hypertensive patients with LVH and HF, followed by those with LVH without HF and patients with HNT without LVH compared to normotensive controls [115]. Plasma CT-1 has lower specificity than plasma NT-proBNP, but has been associated with the progression of HF in patients with HTN [81]. Furthermore, CT-1 has a sensitivity of 70% and specificity of 75% in detecting LVH, as assessed by echocardiography in hypertensive patients, and has been found to be increased in hypertensive patients compared to normotensive subjects [8,116]. For predicting LVH regression in patients with mild to moderate HTN, the sensitivity and specificity of CT-1 were 65 and 96%, respectively, which would be especially helpful in identifying patient at low risk for HHD progression [117]. But the role of CT-1 in the development and maintenance of LVH in experimental hypertensive animal models is controversial [81].

Thus, CT-1 may serve as a biomarker for the severity of heart disease in hypertensive patients, but data remain insufficient to date for routine clinical use [115].

#### 3.2.8. Fibroblast Growth Factor-21

Fibroblast growth factors are a family of signaling proteins, of which fibroblast growth factor (FGF) 21 is a metabolic hormone regulating energy homeostasis. In adult humans, elevated circulating FGF21 was found to be associated with abnormal glucose metabolism, insulin resistance, and dyslipidemia in adults [84]. The Baltimore Longitudinal Study of Aging (BLSA), a cross-sectional observational study, showed that serum FGF21 levels are independently associated with HTN in community-dwelling adults [84]. Similar results have been obtained in other studies showing that elevated circulating levels of FGF21 are significantly associated with elevated BP in animal models or hypertensive patients [84,118].

To date, there are no recommendations to measure FGF21 routinely in the evaluation of essential HTN or HHD.

#### 3.2.9. Klotho

The aging-suppressor gene was first identified in mice in 1997 by Kuro-o et al., and was named Klotho [119]. There are two forms of Klotho, membrane and secreted, which have different functions [85]. Membrane Klotho acts as an obligate co-receptor for FGF-23, which induces phosphate excretion into the urine. Secreted klotho is involved in the regulation of endothelial NO production, renal calcium homeostasis and the inhibition of intracellular insulin and insulin-like growth factor-1 signaling [85]. In humans, the secreted form of Klotho is more dominant than the membrane form [85].

Klotho may be of particular interest as a risk factor for CV disease in humans, as atherosclerosis, oxidative stress, and endothelial dysfunction have been shown to be associated with Klotho expression levels in mice [85,120]. Plasma Klotho was studied by Semba et al. in the Invecchiare in Chianti, “Aging in the Chianti Area” (InCHIANTI), a large, population-based study of aging. The authors showed that plasma Klotho is independently associated with CVD in adults such that adults with higher plasma Klotho levels had a lower risk of CVD [85]. Klotho deficiency was shown to cause endothelial dysfunction and arterial stiffening, as well as HTN and impaired angiogenesis, and may be a predictor of atherosclerosis in humans [121,122]. Serum Klotho levels were 45% lower in untreated patients with HTN and increased vascular rigidity than in healthy participants [123].

A line of experiments has shown an influence of Klotho on many determinants of BP, including, e.g., the LV myocardium, the evolution of LVH, and the RAAS and its components [121,124,125]. 

To date, there has been no role for Klotho testing in daily clinical practice, and future studies are needed to determine and validate its role in the contexts of HTN and HHD in the future.

#### 3.2.10. Practical Implications and Current State of Recommendations for the Biomarker Use 

The identification of biomarkers with potential usefulness for the clinical handling of cardiac diseases has been a field of intensive research during the last few decades. Circulating biomarkers can be applicable in such clinical areas: screening, diagnosis, prognostication, prediction of disease recurrence, and therapeutic monitoring [126]. A number of circulating cardiovascular biomarkers have been shown, with varying levels of evidence, to be associated with the risk of adverse cardiovascular outcomes or to be of value in the management of patients with HHD, but none have shown sufficient diagnostic or prognostic accuracy to be clearly recommended for these purposes. To date, there is no clear recommendation in international guidelines for the implementation of biomarkers in routine clinical practice in patients with arterial hypertension as well as HHD [51,127]. The main barriers for this are considered physiological or pathological conditions, such as age, weight, renal insufficiency, and the severity of the cardiac dysfunction, which may lead to an elevation or reduction in the levels of biomarkers that could influence their diagnostic and prognostic accuracy. 

Over the last few decades, research efforts have resulted in major advances in our understanding of the important role of BNP in the diagnosis and treatment of acute and chronic HF, which has been reflected in European and American Guidelines [128,129]. According to these guidelines, plasma levels of BNP and NT-proBNP are recommended as initial diagnostic tests in patients with symptoms suggestive of HF to exclude or support the diagnosis, are useful for risk stratification, and may guide further cardiac investigation and HF treatment control [128,129,130]. Thus, against the background of well-designed studies, it is now recommended that the upper limits of normal in the non-acute setting be set as 35 pg/mL for BNP and 125 pg/mL for NT-proBNP, and for acute HF (AHF) the cut-offs should be established as <100 pg/mL for BNP and <300 pg/mL for NT-proBNP [128,131,132]. Moreover, The Heart Failure Association of the European Society of Cardiology, in its practical guidance on the use of natriuretic peptide concentrations to improve their diagnostic value, provided three cut-off levels: CHF/AHF unlikely, “Grey Zone”, CHF/AHF likely [57]. 

In our opinion, BNP is the most promising diagnostic and prognostic biomarker for HHD, especially when used to detect subclinical changes and especially stage B pre-heart failure according to the universal definition and classification of heart failure 2021 [130].

Thus, BNP acts as a helpful biomarker for HTN. Its performance, together with the ease of measurement, low cost, and widespread availability, should prompt a wider use of this marker being used for risk stratification in HHD, especially when echocardiography is not available. However, BNP testing and echocardiography are complementary—the combination of both has the potential to increase the diagnosis of early remodeling, as well as early filling pressure increases in HHD. There is also hope that the use of biomarkers, especially BNP, will make it possible to individualize treatment. However, to date, there has been a lack of experimental evidence supporting this diagnostic and personalized approach in arterial hypertension, and we hope that current large RCTs, such as the coArtHA trial, aiming to identify the most effective treatment strategies to control arterial hypertension in sub-Saharan Africa and including focused echocardiography and BNP testing in untreated and clinically uncomplicated arterial hypertension, will allow us to obtain a database that can help us to implement BNP testing in clinical practice [133]. 

Finally, we consider that rather than using the biomarkers individually, the combined assessment of more than one biomarker could have more diagnostic power and may predict HTN and HHD with better sensitivity and specificity [49,77]. We hope that new studies will be performed in the near future to verify all these hypothetical strategies and to better characterize and understand the relationship between circulating cardiac biomarkers and HHD. 

## 4. Suggested Updated Definition and Clinical Classification Matrix of HHD

The global health burden of HTN and HHD has increased significantly during the last decades, which is a major public health issue worldwide—despite the presence of a large armamentarium of effective and well-tolerated drugs, as well as non-drug therapies. 

Despite intensive scientific work, many issues regarding the pathogenesis and factors for the progression of different phenotypes of HHD and HF remain unclear. Moreover, the progression from HTN to HF is, to date, more or less unpredictable, occurring in some patients as maladaptive remodeling or as compensatory remodeling, and even in patients with normal LV function and geometry [50]. Additionally, the mechanisms involved in the transition to HF in HHD are still largely uncertain.

It is well known that many diseases pass through certain stages during their development as a biological continuum. HHD is no exception in this aspect. Different definitions, classifications and methods for the stratification of HHD affecting various aspects (e.g., issues of pathogenesis, stages of development, clinical presentation) have been proposed [1]. However, until now, and in contrast to other important CVDs like myocardial infarction or HF, there has been no universal, generally accepted definition or classification including the pathophysiological and clinical evidence available. This unnecessarily complicates the handling of HHD in clinical practice, but also in clinical trials, as the lack of a uniform definition and classification results in an avoidably large heterogeneity between studies. Furthermore, in clinical practice, HHD is often reduced to the presence of LVH, which does not do justice to the complex disease pattern. 

Taking into account the previous definitions and classifications, as well as the recent years’ findings, we have attempted to summarize the above information and propose a definition and clinical matrix classification of HHD.

Definition:

The term “hypertensive heart disease” describes the complex micro- and macrostructural and functional adaptations of the various parts of the cardiac system to chronically elevated BP. These include the myocardial structure of ventricles and atria, the neurohumoral system, the myocardial vasculature, as well as the electrophysiological properties. These changes characterize the biological continuum from early maladaptive processes to late clinical manifestations. 

Our proposed clinical matrix (Table 2) brings together the extent of the different anatomical and functional changes of atrial and ventricular myocardium, electrophysiological properties and vasculopathy with their clinical significance, and is based on the classification proposed by experts of the Spanish Society of Cardiology (“VIA”: ventricular, ischemia, atrial fibrillation) in 2009 [134]. The scoring was based on the severity of involvement of these selected categories. We have tried to present it in such a manner that it could be easily transferred into clinical practice to give, in brief, a broad picture of the status of HHD in an individual patient with HTN. Taking into account the intensive progress and evolution of modern techniques and biochemical methods in clinical practice, we have included some options that still have practical limitations, but may enter practice in the near future (Table 2).

Similar to many other studies, our review, proposed definitions and clinical matrix have some limitations. Despite our extensive efforts to identify data, in the absence of a generally accepted terminology that overlaps with used terms, we were forced to refer to scientific works that include both HHD and essential hypertension. How the different topics could and should be weighted in terms of risk stratification and prognostic significance remains to be determined. 

## Figures and Tables

**Table 2:** Matrix of hypertensive heart disease.

Clinical Stages	Index	Affected Parts of the Heart			
		Atrium	Ventricle	Vessels	Electrophysiological Properties
Normal	0	No changes ^a^	No evidence of LVH ^a^ Normal LVEF ^a^ No evidence of fibrosis *	No changes ^a,b,^*	No changes
Subclinical manifestation Patients without clinical symptoms/signs but with evidence of structural changes related to hypertension	1	LAVI increased	LVH ^a^ Increased natriuretic peptide levels Evidence of increased filling pressures Evidence of myocardial fibrosis *	Increased Coronary Artery Calcium Score Evidence of atherosclerosis with <70% stenosis Evidence of microvascular dysfunction *	Premature heart beats Conduction disturbances Atrial runs Atrioventricular block IIa-III
Symptomatic manifestation Patients with current or previous symptoms/signs and evidence of structural changes related to hypertension	2		HFpEF HFmrEF HFrEF	Evidence of CAD Evidence of microvascular dysfunction * Dilatation/aneurysm of proximal aorta	Symptomatic changes of 1° Atrial fibrillation Atrioventricular block IIb-III
Major cardiovascular events/secondary prevention	3		AHF	Myocardial infarction Revascularisation	Sudden cardiac death

Note: in 1–3 index—terms according to the approved classifications. ^a^—measured by ECG or Echo, or MRI by standard methods. ^b^—by angiography standard methods. *—in case of advanced imaging. AHF—acute heart failure; BNP—brain natriuretic peptide; CAD—coronary artery disease, LAVI—left atrium volume index; LVEF—left ventricular ejection fraction; LVH—left ventricular hypertrophy; HFmrEF—heart failure with mildly reduced left ventricular ejection fraction; HFpEF—heart failure with preserved left ventricular ejection fraction; HFrEF—heart failure HF with reduced left ventricular ejection fraction.

## Data Availability

Not applicable.
